# Effects of Early Resveratrol Intervention on Skeletal Muscle Mitochondrial Function and Redox Status in Neonatal Piglets with or without Intrauterine Growth Retardation

**DOI:** 10.1155/2020/4858975

**Published:** 2020-05-22

**Authors:** Kang Cheng, Ting Wang, Simian Li, Zhihua Song, Hao Zhang, Lili Zhang, Tian Wang

**Affiliations:** College of Animal Science and Technology, Nanjing Agricultural University, Nanjing 210095, China

## Abstract

Skeletal muscle mitochondrial malfunction of offspring induced by intrauterine growth retardation (IUGR) may be a contributor to growth restriction and metabolic disorder at various periods of life. This study explored the effects of IUGR and resveratrol (RSV) on mitochondrial function and redox status in the longissimus dorsi muscle (LM) of piglets during the sucking period. A total of 36 pairs of IUGR and normal birth weight male piglets were orally fed with either 80 mg RSV/kg body weight/d or 0.5% carboxymethylcellulose sodium during days 7-21 after birth. The results showed that RSV treatment improved anomalous mitochondrial morphology, increased adenosine triphosphate and glycogen contents, and enhanced nicotinamide adenine dinucleotide/reduced form of nicotinamide-adenine dinucleotide ratio in the LM of IUGR piglets. Moreover, the IUGR-induced increased malondialdehyde and protein carbonyl concentrations, abnormal mtDNA number, and suppressed genes expression of mitochondrial biogenesis such as nuclear respiratory factor 1, estrogen-related receptor alpha, and polymerase gamma in the LM were restored to some extent by RSV treatment. Additionally, RSV increased mitochondrial complex V activity in the LM of piglets. Collectively, RSV administration alleviated the LM mitochondrial dysfunction and oxidative damage of IUGR piglets.

## 1. Introduction

Offspring affected by a low birth weight (LBW) after suffering processes of intrauterine growth retardation (IUGR) is receiving increased attention in both human medicine and animals production, owing to the short-term (incremental morbidity and mortality of neonates) and long-term repercussions of LBW (decreased growth patterns, health status, and performance of individuals) [1]. The main reason for IUGR is the decrease of nutrients and oxygen to the fetus by the placenta [2]. In this case, the fetus preferentially shunts blood to the vital organs at the expense of nutrients and oxygen delivery to the periphery [3]. Compared with the brain and heart, skeletal muscle has a lower priority for nutrients redistribution, which makes it particularly susceptible to the nutritional deficiency in *utero* [4]. Epidemiological and animal studies have found that IUGR offspring exhibited impaired skeletal muscle growth and development as well as abnormal glucose metabolism [5–7], which may be associated with a mitochondrial malfunction in skeletal muscle. Mitochondria, “the powerhouse of the cell”, generate energy (i.e., adenosine triphosphate (ATP)) via the efficient electron transport chain (ETC) system for muscular growth and glycogen storage. In addition, mitochondria are the primary site for reactive oxygen species production and are vulnerable to oxidative stress (OS). Several investigations in juvenile rats demonstrated that IUGR reduced mitochondrial nicotinamide adenine dinucleotide (NAD^+^)/reduced form of nicotinamide-adenine dinucleotide (NADH) ratio and ATP synthesis in skeletal muscle, and altered skeletal muscle mitochondrial genes expression (e.g., NADH-ubiquinone-oxidoreductase subunit 4 L) [6, 8]. However, limited information about skeletal muscle mitochondrial redox status in IUGR offspring was available, which needs to be investigated in the present study. But maintaining optimal redox status is essential for mitochondrial function [9]. Therefore, improving the mitochondrial function and redox status in skeletal muscle may be a potential strategy to alleviate the negative effects of IUGR on growth and metabolism.

Resveratrol (trans-3,4′,5-trihydroxystilbene, RSV), a polyphenol isolated from plants such as grapes, peanut, and polygonum cuspidatum, has been described as exhibiting pleiotropic functions including antioxidative and anti-inflammatory properties [10, 11], antiobesogenic [12, 13], antiatherosclerotic [14], and anticancer [15] activities. Previous studies conducted in rodents model demonstrated that RSV can govern glucose homeostasis under high-fat-diet-induced insulin resistance condition by improving mitochondrial ETC complexes activities and antioxidant function, and regulating the genes expression of mitochondrial biogenesis in skeletal muscle [16, 17]. In addition, a recent report showed that RSV improved muscle atrophy in streptozocin-induced diabetic mice evidenced by enhanced muscular mass and function as well as increased mitochondrial contents in part via increasing mitochondrial biogenesis [18]. However, the efficacy of RSV treatment in skeletal muscle mitochondrial dysfunction of IUGR offspring has not yet to be explored. Pigs have been selected as an ideal biomedical model for the study of the occurrence and consequence of IUGR in humans due to the similar morphology, physiology, metabolism, and proportional organ sizes between pigs and humans [19]. Therefore, in the present study, we investigated whether early RSV intervention could improve skeletal muscle mitochondrial function and redox status in neonates with IUGR using a piglet model.

## 2. Materials and Methods

The experiment performed in the present study was approved by the Animal Care and Use Committee of Nanjing Agricultural University. Resveratrol (purity 98%) was purchased from Zhejiang Yixin Pharmaceutical Co., Ltd. (Yixin, China). Carboxymethylcellulose sodium (CMC-Na) was purchased from Sinopharm Chemical Reagent Co., Ltd. (Shanghai, China).

### 2.1. Animals and Experimental Design

During the preparation, healthy sows (Landrace × Yorkshire) with the same parity of the third and similar expected days of farrowing (<3 d) were chosen. At birth, sows that had similar litter sizes (i.e., 11–13 piglets) and met the selection criteria for IUGR piglets were chosen. A total of 72 male newborn piglets (Duroc × (Landrace × Yorkshire)) were collected and tagged from 36 litters (1 normal birth weight (NBW) piglet and 1 IUGR piglet from each litter) for the experiment: 36 were NBW piglets (~1.72 kg) and the other 36 were naturally occurring IUGR littermates (~0.88 kg) according to their birth weight using our previous method [20]. An IUGR piglet was defined as having a birth weight was 2 SD below the mean BW of the total population, whereas a NBW littermate had a birth weight within 0.5 SD unit of the mean birth weight of the whole litter. The NBW and IUGR piglets were cross-fostered after birth by 24 four-parity sows (standardized litter size: 3 experimental piglets and 8 same type nonexperimental piglets). During the sucking period, all piglets remained with sows housed in lactation crates in environmentally controlled rooms. Sows were fed a commercial diet and provided water *ad libitum*. The sucking piglets had no access to the sow's diet. At 7 days of age, the NBW and IUGR piglets were orally fed with 80 mg RSV/kg body weight/d (diluted in 0.5% CMC-Na) or the same volume of 0.5% CMC-Na (diluted in 0.86% saline) for a period of 14 days, respectively. Therefore, all piglets were assigned into 4 groups (six replicates (pens or litters) per group, three piglets per replicate): NBW-CON, NBW-RSV, IUGR-CON, and IUGR-RSV.

### 2.2. Sample Collection

At the end of this experiment, six piglets per group (i.e., one piglet per replicate) were killed according to a previous study [21] and followed by a quick dissection of longissimus dorsi muscle (LM). The LM samples from the left half of the carcass were rapidly collected at the level of the twelfth/thirteenth ribs and cooled using liquid nitrogen and stored at −80°C until subsequent analysis. The remaining half and the contralateral LM samples were collected for the analysis of ultrastructure and mitochondrial parameters.

### 2.3. Transmission Electron Microscopy

For ultrastructural analysis, fresh LM was cut into small pieces and fixed in 2.5% glutaraldehyde (pH 7.4, 0.1 mol/L sodium cacodylate buffer) and 1% osmium tetroxide, dehydrated, and embedded in epoxy resin. The ultrastructure of LM was examined with a transmission electron microscope (Hitachi H-7650). Considering the costs and aim of the experiment, samples from NBW-CON, IUGR-CON, and IUGR-RSV groups were treated and analysed.

### 2.4. Analysis of Skeletal Muscle Metabolites

Glycogen contents in the LM were measured by a commercial kit (Nanjing Jiancheng Institute of Bioengineering, Nanjing, China) according to the anthracenone method. Amounts of ATP in the LM were determined through an ATP Content Assay Kit (Solarbio, Beijing, China) following the manufacturer's instructions. The concentrations of NAD^+^ and NADH in the LM were determined using commercial kits according to the manufacturer's instructions (SinoBestBio, Shanghai, China). The relative intracellular glycogen, ATP, NAD^+^, and NADH levels were calculated based on the quantified concentrations normalized to the weight of the sample.

### 2.5. Mitochondrial Redox Status

The activities of manganese superoxide dismutase (Mn-SOD), glutathione peroxidase (GPX), glutathione reductase (GR) and the concentrations of glutathione (GSH), malondialdehyde (MDA), and protein carbonyl (PC) in the LM mitochondria were determined using commercial kits (Nanjing Jiancheng Institute of Bioengineering, Nanjing, China). All the results were normalized against protein content in each sample for intersample comparison.

### 2.6. Mitochondrial ETC Complexes Activities

The activities of ETC complexes I, II, III, IV, and V were determined with commercially available kits obtained from SinoBestBio (Shanghai, China) following the guidelines of the manufacturer.

### 2.7. Total RNA Isolation and qRT-PCR Assays

Total RNA isolation and qRT-PCR analysis were conducted as described previously [10–13, 22]. Briefly, RNA was isolated from LM samples using Trizol reagent (TaKaRa Biotechnology Co. Ltd., Dalian, China). The quality of RNA was measured by checking its integrity using agarose gel electrophoresis and by conforming that the A260 nm/A280 nm absorbance ratio was between 1.8 and 2.0. After quantified, total RNA (1 *μ*g) was reverse-transcribed into complementary DNA (cDNA) using the PrimeScript™ RT Reagent Kit (TaKaRa Biotechnology Co. Ltd., Dalian, China). The cDNA was amplified via qRT-PCR using ChamQ SYBR qPCR Master Mix Kit (Vazyme, Nanjing, China) and the QuantStudio®5real-time PCR Design & Analysis system (Applied Biosystems, USA). The reaction mixture was prepared using 2 *μ*L of cDNA, 0.4 *μ*L of forward primer, 0.4 *μ*L of reverse primer, 10 *μ*L of ChamQ SYBR qPCR Master Mix (Vazyme, Nanjing, China), 0.4 *μ*L of ROX Reference Dye (Vazyme, Nanjing, China), and 6.8 *μ*L of double-distilled water. The PCR consisted of a prerun at 95°C for 30 s and 40 cycles of denaturation at 95°C for 5 s, followed by a 60°C annealing step for 30 s. The conditions of the melting curve analysis were as follows: one cycle of denaturation at 95°C for 10 s, followed by an increase in temperature from 65 to 95°C at a rate of 0.5°C/s. Melting curve analysis was performed to validate the specificity of the PCR-amplified product. All primers for qRT-PCR were presented in Supplementary Table 1. The relative levels of mRNA expression were calculated using the 2^−*ΔΔ*Ct^ method normalizing against the reference gene *β*-actin expression [23]. The values of the NBW-CON group were used as a calibrator.

### 2.8. Analysis of Skeletal Muscle Mitochondrial DNA (mtDNA) Copy Number

Total genomic DNA was extracted from snap-frozen LM using a universal Genomic DNA Extraction Kit (TSINKE, Beijing, China). The concentration of DNA was quantified and diluted to the same concentration for further analysis. The relative mtDNA content was measured by coamplifying the mt D-loop and the nuclear-encoded *β*-actin gene using qRT-PCR assay. Primer sequences are presented in Supplementary Table 1. The PCR amplification was performed under the same condition as described above. The relative quantification values were calculated according to the 2^-*ΔΔ*Ct^ method [23].

### 2.9. Statistical Analysis

Data were analysed by ANOVA using a 2 × 2 factorial arrangement of treatments with the general linear model procedure (SPSS 22.0; IBM-SPSS Inc., Chicago, IL, USA) and GraphPad Prism (version 5.0, GraphPad Software Inc., San Diego, CA, USA), with the individual piglet as the experimental unit. The statistical model included the effects of BW and RSV and their interaction. When the interaction was significant, the data were reanalysed by one-way analysis of variance and Tukey's post hoc test. The statistical results were presented by mean values and the standard error. Differences were considered significant if *P* < 0.05, and 0.05 < *P* < 0.10 was considered a trend.

## 3. Results

### 3.1. Skeletal Muscle Mitochondrial Ultrastructure

**Figure 1:**
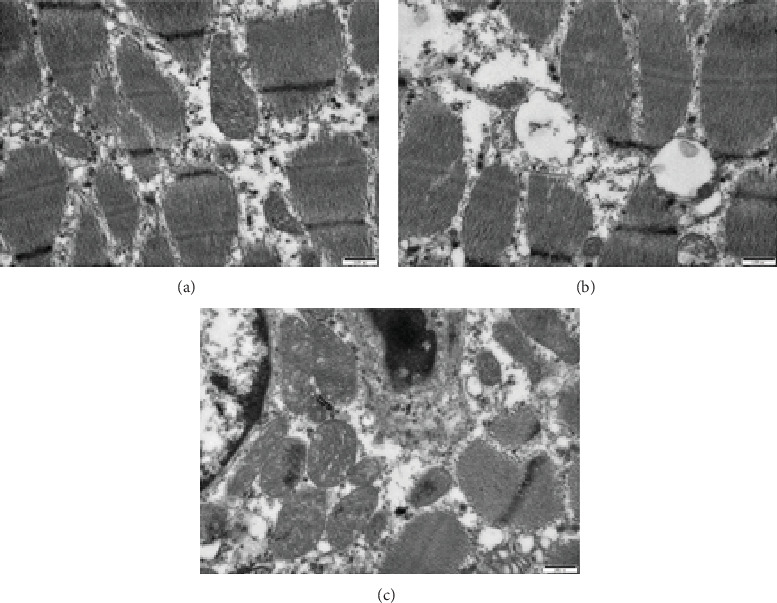
The ultrastructure of longissimus dorsi muscle in piglets at 21 days of age. (a) NBW-CON: normal birth weight piglets were orally fed with 0.5% carboxymethylcellulose sodium. (b) IUGR-CON: intrauterine growth-retarded piglets were orally fed with 0.5% carboxymethylcellulose sodium. (c) IUGR-RSV: intrauterine growth-retarded piglets were orally fed with 80 mg resveratrol/kg body weight/d. Scale bars, 500 nm.

As shown in Figure 1, piglets of the IUGR-CON group exhibited swollen mitochondria with disorganized and fragmented cristae in the LM relative to the NBW-CON group. Expectedly, RSV conferred substantial rescue of the mitochondrial morphological defects in the LM of IUGR-CON piglets.

### 3.2. Skeletal Muscle Metabolites

**Figure 2:**
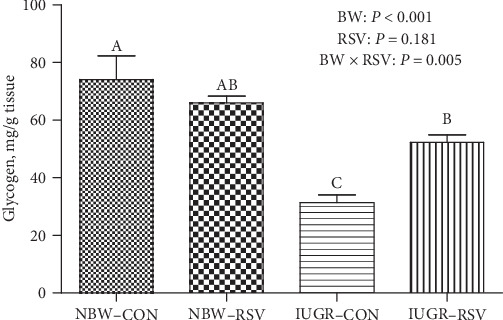
The glycogen content of longissimus dorsi muscle in piglets at 21 days of age. BW: birth weight; RSV: resveratrol; NBW-CON: normal birth weight piglets were orally fed with 0.5% carboxymethylcellulose sodium; NBW-RSV: normal birth weight piglets were orally fed with 80 mg resveratrol/kg body weight/d; IUGR-CON: intrauterine growth-retarded piglets were orally fed with 0.5% carboxymethylcellulose sodium; IUGR-RSV: intrauterine growth-retarded piglets were orally fed with 80 mg resveratrol/kg body weight/d. Results are expressed as mean ± standard error, *n* = 6. Mean Values with unlike superscript were significantly different (*P* < 0.05).

**Figure 3:**
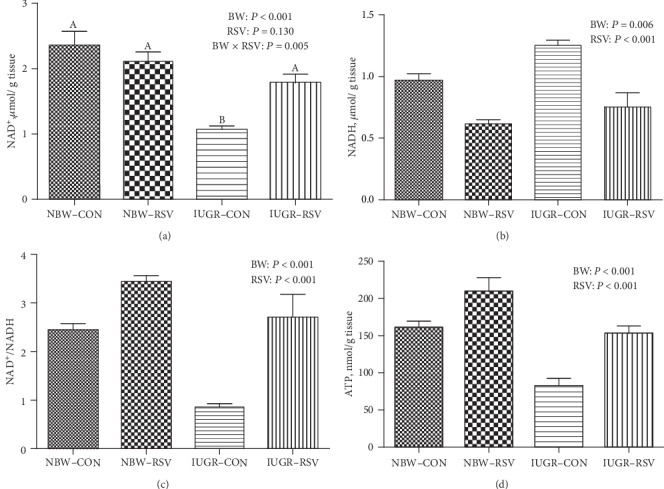
The contents of oxidized form of nicotinamide adenine dinucleotide (NAD^+^, a), reduced form of nicotinamide-adenine dinucleotide (NADH, b) and adenosine triphosphate (ATP, d), and NAD^+^/NADH (c) of longissimus dorsi muscle in piglets at 21 days of age. BW: birth weight; RSV: resveratrol; NBW-CON: normal birth weight piglets were orally fed with 0.5% carboxymethylcellulose sodium; NBW-RSV: normal birth weight piglets were orally fed with 80 mg resveratrol/kg body weight/d; IUGR-CON: intrauterine growth-retarded piglets were orally fed with 0.5% carboxymethylcellulose sodium; IUGR-RSV: intrauterine growth-retarded piglets were orally fed with 80 mg resveratrol/kg body weight/d. Results are expressed as mean ± standard error, *n* = 6. Mean Values with unlike superscript were significantly different (*P* < 0.05).

Compared with the NBW, IUGR pups had lower glycogen level in the LM (*P* < 0.05, Figure 2). Additionally, piglets suffering from IUGR exhibited the diminished levels of ATP and NAD^+^, reduced NAD^+^/NADH ratio, and increased NADH content in the LM (*P* < 0.05, Figure 3). As expected, the amount of ATP and NAD^+^/NADH ratio were significantly increased, and the NADH level was obviously reduced in the LM of piglets due to oral RSV administration (*P* < 0.05). A noticeable interaction between BW and RSV treatment was observed in the levels of glycogen and NAD^+^ (*P* < 0.05); the IUGR-induced decreased glycogen and NAD^+^ concentrations in the LM of piglets were restored by RSV supplementation (*P* < 0.05).

### 3.3. Skeletal Muscle mtDNA Content

**Figure 4:**
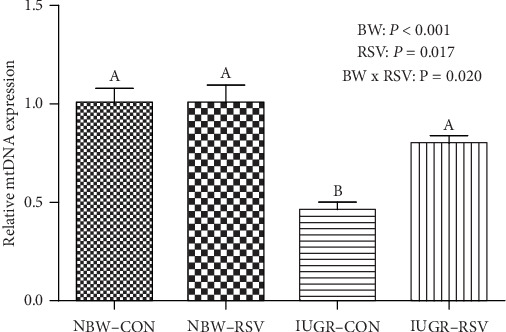
The mitochondrial DNA (mtDNA) content of longissimus dorsi muscle in piglets at 21 days of age. BW: birth weight; RSV: resveratrol; NBW-CON: normal birth weight piglets were orally fed with 0.5% carboxymethylcellulose sodium; NBW-RSV: normal birth weight piglets were orally fed with 80 mg resveratrol/kg body weight/d; IUGR-CON: intrauterine growth-retarded piglets were orally fed with 0.5% carboxymethylcellulose sodium; IUGR-RSV: intrauterine growth-retarded piglets were orally fed with 80 mg resveratrol/kg body weight/d. Results are expressed as mean ± standard error, *n* = 6. Mean Values with unlike superscript were significantly different (*P* < 0.05).

As shown in Figure 4, in the LM, a significant interaction effect between BW and RSV was observed in mtDNA copy number (*P* < 0.05); RSV alleviated the diminished mtDNA content induced by IUGR (*P* < 0.05).

### 3.4. Skeletal Muscle Mitochondrial Redox Status

**Figure 5:**
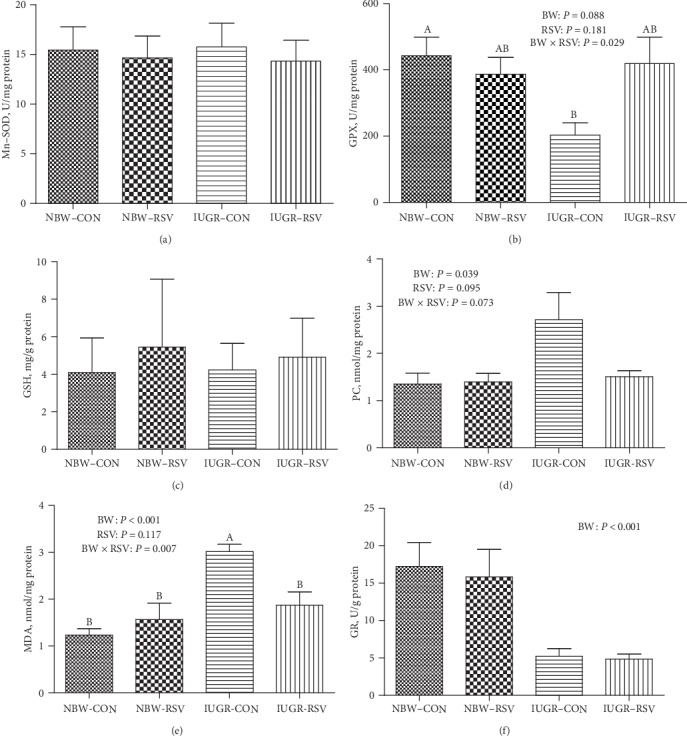
The mitochondrial redox status of longissimus dorsi muscle in piglets at 21 days of age. (a) Mn-SOD: manganese superoxide dismutase; (b) GPX: glutathione peroxidase; (c) GSH: glutathione; (d) PC: protein carbonyl; (e) MDA: malondialdehyde; (f) GR: glutathione reductase. BW: birth weight; RSV: resveratrol; NBW-CON: normal birth weight piglets were orally fed with 0.5% carboxymethylcellulose sodium; NBW-RSV: normal birth weight piglets were orally fed with 80 mg resveratrol/kg body weight/d; IUGR-CON: intrauterine growth-retarded piglets were orally fed with 0.5% carboxymethylcellulose sodium; IUGR-RSV: intrauterine growth-retarded piglets were orally fed with 80 mg resveratrol/kg body weight/d. Results are expressed as mean ± standard error, *n* = 6. Mean Values with unlike superscript were significantly different (*P* < 0.05).

As shown in Figure 5, compared with the NBW, IUGR piglets exhibited mitochondrial OS in the LM evidenced by the increased MDA (*P* < 0.05) and PC (*P* < 0.05) levels as well as the reduced activities of GPX (*P* = 0.068) and GR (*P* < 0.05). The amount of mitochondrial PC in the LM of piglets was decreased by RSV treatment (*P* = 0.095). An interaction between BW and RSV was observed in GPX activity (*P* < 0.05), and amounts of PC (*P* = 0.073) and MDA (*P* < 0.05) in the LM mitochondria of piglets. Resveratrol suppressed the elevated MDA contents in the LM mitochondria induced by IUGR (*P* < 0.05). However, no differences were found in the LM mitochondrial Mn-SOD activity and GSH level of piglets among 4 groups (*P* > 0.05).

### 3.5. Skeletal Muscle Mitochondrial ETC Complexes Activities

**Figure 6:**
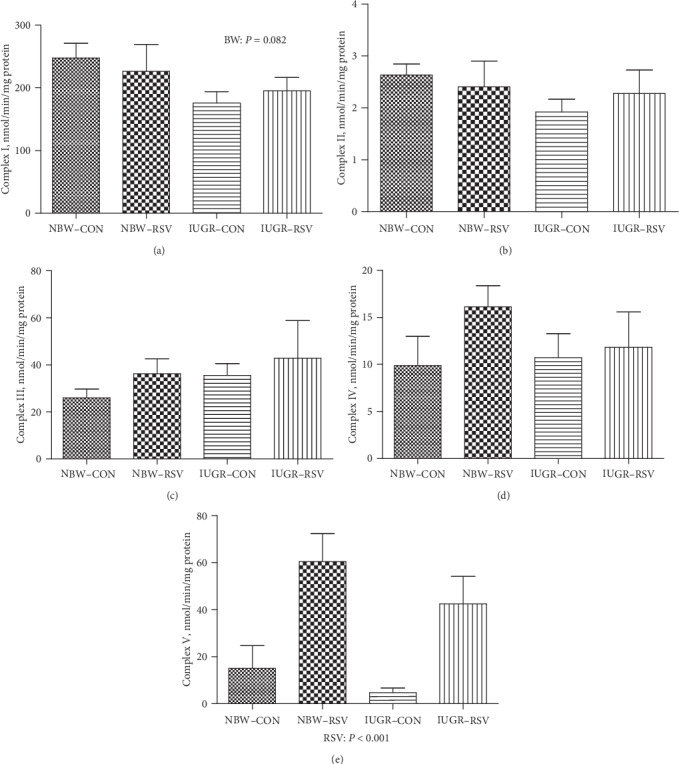
The mitochondrial respiratory chain complexes I (a), II (b), III (c), IV (d), and V (e) activities of longissimus dorsi muscle in piglets at 21 days of age. BW: birth weight; RSV: resveratrol; NBW-CON: normal birth weight piglets were orally fed with 0.5% carboxymethylcellulose sodium; NBW-RSV: normal birth weight piglets were orally fed with 80 mg resveratrol/kg body weight/d; IUGR-CON: intrauterine growth-retarded piglets were orally fed with 0.5% carboxymethylcellulose sodium; IUGR-RSV: intrauterine growth-retarded piglets were orally fed with 80 mg resveratrol/kg body weight/d. Results are expressed as mean ± standard error, *n* = 6. Mean Values with unlike superscript were significantly different (*P* < 0.05).

The effects of BW and RSV on mitochondrial ETC complexes activities in the LM of piglets was shown in Figure 6. Compared with the NBW, IUGR resulted in the decreased complex I activity in the LM mitochondria of piglets (*P* = 0.082). Resveratrol elevated the activity of complex V in the LM mitochondria of piglets (*P* < 0.05). However, the activities of complex II, III, and IV in the LM mitochondria were not affected by BW or/and RSV (*P* > 0.05).

### 3.6. Skeletal Muscle Genes Expression Related to Mitochondrial Biogenesis

**Figure 7:**
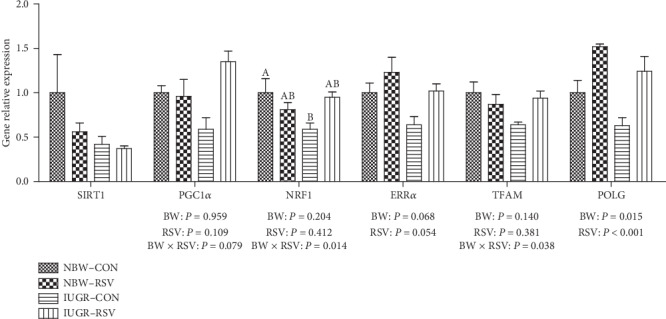
The genes expression related to mitochondrial biogenesis of longissimus dorsi muscle in piglets at 21 days of age. BW: birth weight; RSV: resveratrol; NBW-CON: normal birth weight piglets were orally fed with 0.5% carboxymethylcellulose sodium; NBW-RSV: normal birth weight piglets were orally fed with 80 mg resveratrol/kg body weight/d; IUGR-CON: intrauterine growth-retarded piglets were orally fed with 0.5% carboxymethylcellulose sodium; IUGR-RSV: intrauterine growth-retarded piglets were orally fed with 80 mg resveratrol/kg body weight/d. Results are expressed as mean ± standard error, *n* = 5. Mean Values with unlike superscript were significantly different (*P* < 0.05).

The effects of BW and RSV on the mRNA expression of mitochondrial biogenesis in the LM of piglets was shown in Figure 7. The profile of estrogen-related receptor alpha (ERR*α*, *P* = 0.068) and polymerase gamma (POLG, *P* < 0.05) mRNA in the LM piglets was decreased by IUGR. In contrast, RSV increased the mRNA level of ERR*α* (*P* = 0.054) and POLG (*P* < 0.05) in the LM of piglets. An interaction between BW and RSV was found in the mRNA expression of peroxisome proliferation activated receptor gamma coactivator-1 alpha (*P* = 0.079), mitochondrial transcription factor A (TFAM, *P* < 0.05), and nuclear respiratory factor 1 (NRF1, *P* < 0.05). Resveratrol increased (*P* = 0.091) the inhibited expression of NRF1 mRNA in the LM of piglets induced by IUGR. No difference in gene expression of sirtuin 1 was observed in the LM of piglets among these groups (*P* > 0.05).

### 3.7. Skeletal Muscle Genes Expression Related to Energy Metabolism

**Figure 8:**
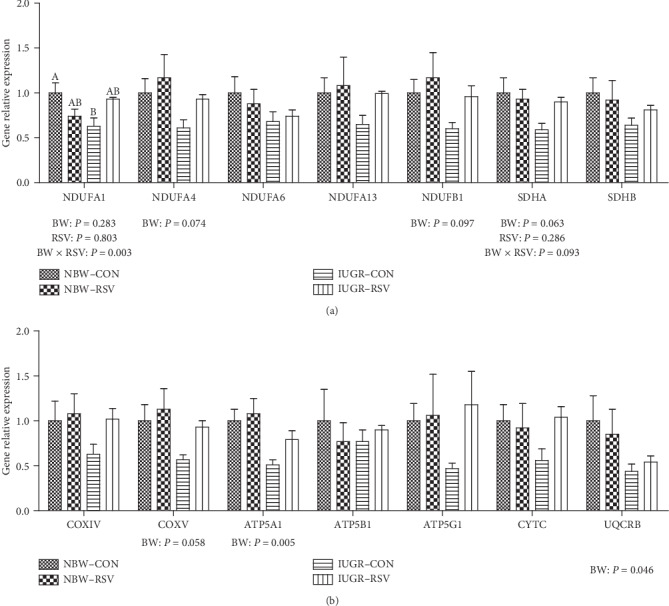
The genes expression related to mitochondrial energy metabolism of longissimus dorsi muscle in piglets at 21 days of age. BW: birth weight; RSV: resveratrol; NBW-CON: normal birth weight piglets were orally fed with 0.5% carboxymethylcellulose sodium; NBW-RSV: normal birth weight piglets were orally fed with 80 mg resveratrol/kg body weight/d; IUGR-CON: intrauterine growth-retarded piglets were orally fed with 0.5% carboxymethylcellulose sodium; IUGR-RSV: intrauterine growth-retarded piglets were orally fed with 80 mg resveratrol/kg body weight/d. Results are expressed as mean ± standard error, *n* = 5. Mean Values with unlike superscript were significantly different (*P* <0.05).

As shown in Figure 8, IUGR led to the reduced gene expression of NADH dehydrogenase (ubiquinone) 1 alpha subcomplex 4 (NDUFA4, *P* = 0.074), NADH dehydrogenase (ubiquinone) 1 beta subcomplex 1 (NDUFB1, *P* = 0.097), succinate dehydrogenase complex flavoprotein subunit A (*P* = 0.063), cytochrome c oxidase V (COXV, *P* = 0.058), ATP synthase alpha subunit (ATP5A1, *P* < 0.05), and ubiquinol-cytochrome c reductase binding protein (UQCRB, *P* < 0.05) in the LM of piglets compared with the NBW. However, there was no significant interaction between the effects of BW and RSV treatment on the mRNA abundance of these genes in the LM, except on NDUFA1 (*P* < 0.05). Resveratrol restored (*P* = 0.074) the suppressed expression of muscular NDUFA1 at the transcriptional level of IUGR piglets. In addition, the gene expression of NDUFA6, NDUFA13, succinate dehydrogenase complex iron sulfur subunit B, COXIV, ATP synthase beta polypeptide, ATP synthase F0 complex subunit C1, and cytochrome C in the LM of piglets was not altered among these groups (*P* > 0.05).

## 4. Discussion

To the best of our knowledge, this is the first study to determine the negative influence of IUGR on the mitochondrial function and redox status in skeletal muscle of neonatal pigs, and the therapeutic effect of RSV. Notably, novel and important findings from the present study are that reduced ATP production, decreased glycogen storage and NAD^+^/NADH ratio, altered mitochondrial biogenesis and redox homeostasis, and diminished ETC complexes activities in skeletal muscle of the sucking IUGR piglets were mitigated to some extent by early RSV intervention.

Skeletal muscle is the main component of total body mass, and it can absorb circulatory glucose and synthesize glycogen, being responsible for metabolic homeostasis [3, 24]. But ATP is necessary for growth and glycogen synthesis, and IUGR animals exhibit reduced postnatal muscular growth [25, 26] and glycogen storage [6, 8]. These findings were corroborated by the present study that IUGR diminished ATP and glycogen contents in the LM of piglets. The deficiency of muscular energy of IUGR piglets in this study may be associated with impaired mitochondrial antioxidant defense system and decreased mitochondrial biogenesis.

In the present study, IUGR piglets had inhibited GR and GPX activities and increased MDA and PC concentrations in the LM mitochondria. MDA and PC are products of lipid and protein oxidation by superfluous free radicals, respectively, all of which are regarded as important markers of OS [27]. The occurrence of OS is due to the fact that the production of free radicals exceeds the scavenging capacity of antioxidants [28]. Both GR and GPX play a critical role in scavenging free radicals. The impairment of antioxidant enzymes activities in this study further confirmed that IUGR led to OS in the LM mitochondria. In the present study, the loss of complex I activity in the LM mitochondria of IUGR could be induced by OS, which could result in a shutdown of mitochondrial energy generation. Similar results were observed in hepatic mitochondria of piglets that IUGR increased MDA content and reduced complex I and III activities due to the increased production of superoxide radicals, and consequently inhibited ATP level [21]. Additionally, the lower NAD^+^/NADH ratio in this study may be explained by the reduced activity of complex I, which couples electron from NADH to quinone with translocation of proton across the inner mitochondrial membrane for ATP production [29], thereby increasing NADH relative to NAD^+^. Moreover, a decrease in NAD^+^/NADH ratio inhibits the flux of glycolytic and tricarboxylic acid cyclic metabolites through the mitochondria, in turn, resulting in reduced ATP production [8]. Thus, in this study, the OS-reduced complex I activity due to impaired antioxidant defense system in the LM mitochondria of IUGR piglets may contribute to muscular energy deficiency.

Mitochondrial ATP production is related to the mitochondrial number, which could be determined quantitatively by mtDNA copy number [30]. Mitochondrial DNA content is regulated by mitochondrial biogenesis [31]. Mitochondrial biogenesis is under the regulation of some transcriptional factors such as NRF1 [32]. In the current study, we also found that IUGR reduced the genes expression of NRF1, ERR*α*, and POLG in the LM, involving in the compromised mtDNA level. The suppression of mitochondrial biogenesis may explain the reduced genes expression of NDUFA1, NADUFA4, NADUFB1, SDHA, UQCRB, COXV, and ATP5A1 in this study. NDUFA1, NADUFA4, and NADUFB1 are key components of complex I, and their inhibited mRNA abundance may be associated with the reduced activity of complex I in this study. Similar results in piglets model have shown that IUGR-diminished hepatic ATP generation is also associated with compromised mitochondrial biogenesis [21, 33]. Therefore, the lower mitochondrial number associated with inhibited mitochondrial biogenesis in the LM of IUGR piglets might also be an important factor leading to the diminished production of ATP in this study.

As expected, in the present study, administration of RSV to IUGR piglets enhanced the synthesis of ATP and glycogen, and normalized NAD^+^/NADH ratio and mtDNA content in skeletal muscle, which directly or indirectly indicated that RSV improved mitochondrial dysfunction and energy deficiency in the LM of IUGR piglets. We found that RSV reduced the elevated MDA and PC concentrations in the LM mitochondria of IUGR piglets but had no effects on antioxidants levels, suggesting that RSV alleviated the LM mitochondrial OS by the compromised free radical levels rather than the enhancement of antioxidant defense system. In this case, on the one hand, RSV can directly scavenge free radicals (e.g., superoxide anion and hydrogen peroxide) due to the presence of phenolic hydroxyl groups [12]; on the other hand, RSV indirectly inhibited the process of free radicals synthesis via enhancing mitochondrial function [21]. In the present study, we have also seen that an increase in complex V or ATP synthase activity in the LM mitochondria of IUGR-RSV piglets playing a major role in bioenergetics, which uses the exergonic proton backflow for ATP synthesis from ADP and inorganic phosphate in the matrix [34]. Of note, in this study, RSV increased the muscular NRF1, ERR*α*, and POLG gene expression of IUGR piglets. NRF1, a nuclear transcriptional factor, regulates the expression of TFAM and other nuclear-encoded mitochondrial subunits of ETC complexes such as ATP synthase. The increase of NRF1 gene expression may be related to the enhanced complex V activity in the present study. As a transcriptional factor, ERR*α* regulates the transcriptional expression of genes required for mitochondrial biogenesis and oxidative phosphorylation. Previous study reported that ERR*α* deficiency exacerbated the cisplatin-induced decrease in mtDNA and altered mitochondrial structure [35]. The POLG gene encodes the mitochondrial DNA polymerase, which is responsible for the replication of the mitochondrial genome. Mutations in POLG can cause early childhood mtDNA depletion syndromes or later-onset syndromes resulting from mtDNA deletions [36]. These results in this study indicated that the LM mitochondrial biogenesis of IUGR piglets was improved due to RSV administration. Similar results in diabetic mice [18] and obesity mice [17] have demonstrated that RSV treatment resulted in an increase in mitochondrial biogenesis in skeletal muscle. Also, Zhang et al. [21] found similar results in hepatic mitochondria of IUGR piglets fed with RSV. In addition, damage to mtDNA could lead to consecutive free radical bursts, which further exacerbated the mitochondrial dysfunction, setting a vicious cycle [37]. Therefore, the therapeutic and beneficial effects of RSV on the LM mitochondria of IUGR piglets may be associated with the improvement of mitochondrial biogenesis and/or diminished amounts of free radicals.

## 5. Conclusions

In conclusion, our results suggested that RSV alleviated the LM mitochondrial dysfunction of IUGR piglets, as evidenced by suppression of MDA and PC overproduction; maintenance of ATP, glycogen, and mtDNA levels; enhancement of ETC complex V activity, and mitochondrial biogenesis. These novel findings provide a new highly promising strategy for preventing the reduced growth of muscle and metabolic diseases of IUGR offspring after birth.

## Data Availability

The data used to support the findings of this study are available from the corresponding authors upon request.

## Supplementary Materials

Supplementary Materials Supplementary Table 1: primer sequences of the target and reference genes.
