# Supplementary material for: Effects of Early Resveratrol Intervention on Skeletal Muscle Mitochondrial Function and Redox Status in Neonatal Piglets with or without Intrauterine Growth Retardation
Source: Oxid Med Cell Longev. 2020 May 22;2020:4858975. doi: 10.1155/2020/4858975 (PMC7261333; doi:10.1155/2020/4858975)
Supplement: Supplementary Materials — Supplementary Table 1: primer sequences of the target and reference genes. [file 4858975.f1.docx]

TABLE 1: Primer sequences of the target and reference genes.

| Gene | GenBank ID | Sequence (5’ → 3’) | Product length (bp) |
| --- | --- | --- | --- |
| SIRT1 | NM_001145750.2 | TTGCAACAGCATCTTGCCTG | 91 |
|  |  | GGACATCGAGGAACCACCTG |  |
| PGC-1α | NM_213963.2 | GCTTGACGAGCGTCATTCAG | 100 |
|  |  | GGTCTTCACCAACCAGAGCA |  |
| NRF1 | XM_021078993.1 | GAAGCTGTCCAGGGGCTTTA | 116 |
|  |  | ATCCATGCTCTGCTACTGGG |  |
| ERRα | NM_001170521.1 | ACGAGTGCGAGATCACCAAG | 130 |
|  |  | TTGTACTTCTGTCGCCCACC |  |
| TFAM | NM_001130211.1 | AGCGAGGTCTGAAGAGTTGC | 114 |
|  |  | TTGCACCCGTAGACAAAGCA |  |
| POLG | XM_001927064.5 | CTGTCAGATGAGGGCGAGTG | 133 |
|  |  | ACTTCTTCCGTCGTGACTTTCT |  |
| NDUFA1 | XM_003135339.5 | GCTTCCGGGGAAGGAATCAA | 101 |
|  |  | CCGGGGAGAATTTCGAACCA |  |
| NDUFA4 | NM_001097468.2 | AACCCTGGAATAAACTGGGTCC | 152 |
|  |  | TGCGGATGGCTTCTGGAAAA |  |
| NDUFA6 | NM_001185178.1 | TCTCAGAGCCTTGCATGTCG | 85 |
|  |  | AAGCCATCCAGCATCGTACC |  |
| NDUFA13 | NM_001244646.1 | ATGAAGGATGTGCCGGACTG | 125 |
|  |  | CCATAGGTGGCGCTGAGAAT |  |
| NDUFB1 | XM_003482306.4 | TGCCTTCCGGAACAAGAGTC | 88 |
|  |  | GCAATTCAGCCACAGCCTTT |  |
| SDHA | XM_021076930.1 | CAATAAGAGGTCGTCGGCCA | 127 |
|  |  | AGAGAGACCAAACGCAGCTC |  |
| SDHB | NM_001104953.1 | TCCTATGGTGTTGGATGCGT | 124 |
|  |  | AGTGTTGCCTCCGTTGATGT |  |
| UQCRB | NM001185172.1 | CATCAGGCAACGCTTCTGTC | 81 |
|  |  | TATACCCTCCAGCCACTTGC |  |
| CytC | NM_001129970.1 | CTGGGGAGAGGAGACACTGAT | 158 |
|  |  | AGGCGGTGGCCAACTTTTAC |  |
| COX IV | XM_021093705.1 | CCAAGTGGGACTACGACAAGAAC | 131 |
|  |  | CCTGCTCGTTTATTAGCACTGG |  |
| COX V | NM_001007517.1 | ATCTGGAGGTGGTGTTCCTACTG | 160 |
|  |  | GTTGGTGATGGAGGGGACTAAA |  |
| ATP5A1 | NM_001185142.1 | ACGCCATTGATGGAAAGGGT | 98 |
|  |  | TGGTTCCCGCACAGAGATTC |  |
| ATP5B1 | XM_001929410.5 | CATGTTGGGCTTTGTGGGTC | 139 |
|  |  | ATAGTCTCTGGCAGGCTGGA |  |
| ATP5G1 | NM_001025218.2 | GTGAGTCAGTCACCTTGAGC | 110 |
|  |  | GAGAAATGAGTAGCGCCCCG |  |
| β-actin | XM_003124280.5 | CTCCAGAGCGCAAGTACTCC | 153 |
|  |  | AATGCAACTAACAGTCCGCC |  |
| mt D-loop | AF276923.1 | GCCTTGCCAAACCCCAAAAA | 137 |
| (mtDNA analysis) |  | TAGGTGCCTGCTTTCGTAGC |  |
| β-actin | DQ452569.1 | GAAGCTCAGTCGGGCTTCTC | 95 |
| (mtDNA analysis) |  | ATGTCGACGTCGCACTTCAT |  |

ATP5A1, ATP synthase alpha subunit; ATP5B, ATP synthase beta polypeptide; ATP5G1, ATP synthase F0 complex subunit C1; β-actin, beta actin; COX IV, cytochrome c oxidase IV; COX V, cytochrome c oxidase V; CytC, cytochrome C; ERRα, estrogen-related receptor alpha; mt D-loop, mitochondrial D-loop; NDUFA1, NADH dehydrogenase (ubiquinone) 1 alpha subcomplex 1; NDUFA4, NADH dehydrogenase (ubiquinone) 1 alpha subcomplex 4; NDUFA6, NADH dehydrogenase (ubiquinone) 1 alpha subcomplex 6; NDUFA13, NADH dehydrogenase (ubiquinone) 1 alpha subcomplex 13; NDUFB1, NADH dehydrogenase (ubiquinone) 1 beta subcomplex 1; NRF1, nuclear respiratory factor 1; POLG, polymerase gamma; PGC-1α, peroxisome proliferation activated receptor gamma coactivator-1 alpha; SDHA, succinate dehydrogenase complex flavoprotein subunit A; SDHB, succinate dehydrogenase complex iron sulfur subunit B; SIRT1, sirtuin 1; TFAM, mitochondrial transcription factor A; UQCRB, ubiquinol-cytochrome c reductase binding protein.
